# Correlating Microstrain and Activated Slip Systems with Mechanical Properties within Rotary Swaged WNiCo Pseudoalloy

**DOI:** 10.3390/ma13010208

**Published:** 2020-01-03

**Authors:** Pavel Strunz, Lenka Kunčická, Přemysl Beran, Radim Kocich, Charles Hervoches

**Affiliations:** 1 Nuclear Physics Institute of the CAS, 250 68 Řež, Czech Republic; pberan@ujf.cas.cz (P.B.); hervoches@ujf.cas.cz (C.H.); 2 Faculty of Materials Science and Technology, VŠB-Technical University of Ostrava, 708 00 Ostrava-Poruba, Czech Republic; lenka.kuncicka@vsb.cz (L.K.); radim.kocich@vsb.cz (R.K.); 3 European Spallation Source ERIC, 225 92 Lund, Sweden

**Keywords:** tungsten, rotary swaging, neutron diffraction, dislocations, microstrain

## Abstract

Due to their superb mechanical properties and high specific mass, tungsten heavy alloys are used in demanding applications, such as kinetic penetrators, gyroscope rotors, or radiation shielding. However, their structure, consisting of hard tungsten particles embedded in a soft matrix, makes the deformation processing a challenging task. This study focused on the characterization of deformation behavior during thermomechanical processing of a WNiCo tungsten heavy alloy (THA) via the method of rotary swaging at various temperatures. Emphasis is given to microstrain development and determination of the activated slip systems and dislocation density via neutron diffraction. The analyses showed that the grains of the NiCo2W matrix refined significantly after the deformation treatments. The microstrain was higher in the cold swaged sample (44.2 × 10^−4^). Both the samples swaged at 20 °C and 900 °C exhibited the activation of edge dislocations with <111> {110} or <110> {111} slip systems, and/or screw dislocations with <110> slip system in the NiCo2W matrix. Dislocation densities were determined and the results were correlated with the final mechanical properties of the swaged bars.

## 1. Introduction

Given their excellent mechanical and physical properties, tungsten heavy alloys (THAs) are popular for demanding applications in the military, radiation shields, and highly demanding components such as aircraft counter-balances and gyroscope rotors. THAs usually consist of 90–97 wt.% of tungsten plus other elements, such as Co, Ni, Fe, and Cu [1]. THAs are generally two-phase composites consisting of spherical tungsten particles/agglomerates surrounded by a ductile matrix [2]. Nevertheless, the structure characteristics consequently impacting the performance of the final product can non-negligibly be altered by even the slightest modifications to the processing technology. By this reason, characterization of the occurring structural phenomena, such as the possible presence of adiabatic shear bands (ASBs), determination of the presence of microstrain and residual stress, and characterization of dislocations and active slip systems are of the utmost importance. The first mentioned phenomenon has been investigated quite thoroughly [3,4,5], but detailed works characterizing other structure phenomena during deformation processing of THAs are scarce [6,7].

Recently, several pieces of research have shown that imposing intensive shear strain into THAs during their production enhances their utility properties and ballistic performance [8,9]. Effective structure refinement can preferably be introduced via methods of severe plastic deformation (SPD), for example via the widely researched equal channel angular pressing (ECAP) and its modifications, which have been proven to effectively refine the grain size down to several hundreds of nanometres for various materials from aluminium to tungsten [10,11,12,13,14,15,16]. Nevertheless, the major drawback of prospective industrial use of SPD methods is their limited applicability for bulk volume samples. For example, the most effective high pressure torsion (HPT) method is only suitable to process coin-like samples [17]. By these reasons, THAs are mostly fabricated via various thermomechanical treatments and more conventional technologies, such as hot extrusion [18,19], cold rolling [20], and swaging [21,22,23].

Rotary swaging (RS) is an intensive plastic deformation method advantageously used in the industry to gradually reduce cross-sections and increase lengths of axisymmetric workpieces [24,25]. Given by its incremental character and dominant compressive stress state, the method can favourably be used to process sintered materials [26]. The dominant shear strain mechanism enables elimination of residual porosity and imparts significant structure refinement.

The primary aim of the presented study was to determine microstrain and characterize the dislocations and active slip systems in the original sintered THA, as well as in the rotary swaged bars, in order to characterize the effects of thermomechanical treatment on the microstructure and final mechanical properties. Throughout the paper, the term “microstrain” denotes the root mean square of the variations in the lattice parameters across the individual crystallites across microscopic distances (root mean square strain, RMSS). In contrast, the term “macrostrain” (not reported here) refers to the overall change in the lattice parameter caused, for example, by a residual stress distribution across the whole component. Generally, microstrain can be caused by a distribution of crystal defects such as vacancies, dislocations, stacking or twin faults.

Neutron powder diffraction method was used as the principal tool for the characterization of structure and microstructure. The strength of neutron diffraction lies in the possibility to provide information from the bulk of the sample, not only from its near-surface region. This fact is very important, especially for THA, where the material is composed mainly of tungsten which is highly absorbing other radiation (X-ray, electrons). When neutrons are used, the signal is averaged over a large volume and the effects of local variability, large grain size and possible local artefacts are minimized.

## 2. Materials and Methods

The W-Ni-Co (93-6-1) wt.% (80.9-16.4-2.7 at.%) pseudo-alloy was produced by powder metallurgy. The particle size of initial W, Ni and Co powders was in the range of 2–4 µm. The weighted mixture of the powders was homogeneously mixed and then sintered at 1500 °C under H_2_ protective atmosphere, and subsequently quenched in water. The as-sintered material, i.e., bars with approximately 12 × 18 mm^2^ elliptical cross sections, is denoted as W_0 throughout the following text.

The sintered bars were further processed by rotary swaging (RS) into circular swaged bars with a diameter of 10 mm. RS was performed in two different ways: at room temperature (sample W_A) and at 900 °C (sample W_B).

The neutron diffraction patterns for structure and microstrain determination were collected at ambient temperature on the MEREDIT diffractometer of CANAM infrastructure at NPI Řež near Prague [27]. A mosaic Cu monochromator (reflection 220) provided neutrons with a wavelength of λ = 1.46 Å. A small (0.4%) λ/2 (0.73 Å) contamination of the incoming beam was present and was taken into account during the analysis. The samples were fixed in the beam using a sample holder enabling a sample rotation along the vertical axis to average the texture and large-grain influence on the diffracted intensities within the diffraction plane. A neutron beam size was selected to submerge the sample fully in the beam. The diffraction patterns were collected from 4 to 144° of 2θ (where θ is the scattering angle) with a step size of 0.08° using a multi-detector bank (35 ^3^He point counters with corresponding 10’ Soller collimators). In order to assess the grain size, an additional neutron diffraction measurement was performed using the TKSN-400 diffractometer [28] equipped with a 2D position-sensitive detector. This measurement was carried out with the neutron wavelength of λ = 1.21 Å.

Further sample analyses were performed using scanning and transmission electron microscopy (SEM and TEM) on ion-polished transversal samples taken from both the swaged pieces and from the original sintered material. SEM-EBSD (electron backscatter diffraction) analyses were performed using a TESCAN Lyra 3 device equipped with NordlysNano EBSD detector with the scan step of 0.1 µm. The substructures and grains analyses were performed using ATEX [29] and Channel 5 software. TEM images were acquired on ion-polished thin foils with a JEOL 2100F device.

The last step was characterization of mechanical properties via tensile tests performed to evaluate the mechanical behavior of the sintered and swaged material states and to determine their ultimate tensile strength (UTS) and maximum elongation. Tensile testing was performed with 100 mm long bars and a strain rate of 1.3 × 10^−3^ s^−1^ using a Zwick device. By the reason that determination of elastic properties on tungsten heavy alloys is complicated by tensile tests due to possible deflections of the stress-strain curves, the elastic moduli were additionally determined via ultrasound measurements by an Olympus 38DL Plus device which applies the Pulse Echo Overlap (PEO).

## 3. Results

### 3.1. Phase Identification and Grain Size

**Figure 1:**
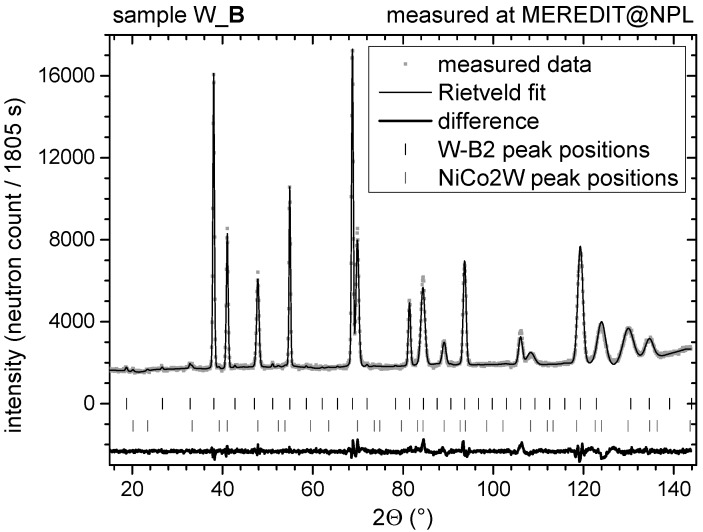
Measured and calculated neutron diffraction pattern for W_B sample used for phase determination as well as for microstrain characterization. The Bragg positions for individual recognized phases (from the top W-B2 and NiCo2W; it should be noted that also the peak positions for λ/2 contamination wavelength are shown) are below the intensity curve. The difference between the measured and calculated intensity is shown as well.

Prior to microstrain determination, phase identification was done using diffractograms measured at neutron diffractometer MEREDIT [27] for all the samples. An example of the measured and calculated neutron diffraction pattern for the sample W_B with recognized phases is shown in Figure 1. The other two samples exhibited similar phase composition and the diffractograms are similar, although they differ in details due to peak broadening, as will be discussed later in the text. The phase identification and analysis in all the samples was performed by full-pattern refinement using FullProf software [30].

Two phases were identified in all samples. The main phase is α-W (B2 structure; within the text, it is referred to as W-B2 phase). The second phase with a weight fraction of 6%–7% has pure-Ni-like structure (fcc) with the lattice parameter of about 3.60 Å. The lattice parameter of this Ni-like phase is slightly larger than the one for pure nickel (3.55 Å), thus indicating alloying with larger W atoms (atomic radius of W, Ni and Co is 139, 124 and 125 pm, respectively). During the structural refinement, it was assumed that the second phase consists of Ni and Co in the same ratio as the initial composition (6:1) with a further addition of 2 at.% of W. This secondary phase is denoted NiCo2W in what follows.

**Figure 2:**
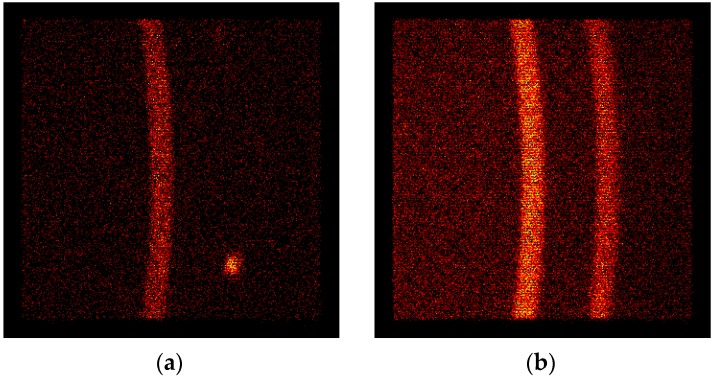
Part of the diffractogram taken with 2D detector at TKSN-400 diffractometer (in the angular range 12.5–22°). (**a**) W_0 sample: Left strip, W-B2 110 reflection; right spot, NiCo2W 111 large-grain reflection; (**b**) W_B sample: The smooth strips of intensities from W-B2 110 (left) and NiCo2W 111 (right) reflections of fine-grained phases after rotary swaging.

It was found using data from 2D detector of TKSN-400 diffractometer [28] that the W_0 bar (i.e., the sample without rotary swaging forming) has a fine-grained microstructure for the W-B2 phase but very raw-grained microstructure of the NiCo2W phase. The NiCo2W phase produces spots on the 2D detector while the W grains of the W-B2 phase result in a smooth Debye–Scherrer diffraction conus, as can be seen in Figure 2a. Taking into account the gauge volume of 0.13 cm^3^, detector characteristics, and the geometrical arrangement of the experiment, the grain size of the NiCo2W phase in the W_0 sample can be estimated to be in the range 0.2–1 mm.

The large-grain microstructure is refined by rotary swaging. W_A and W_B samples already exhibit the fine-grained NiCo2W phase, as can be seen in Figure 2b, taken for the same angular range as Figure 2a. The spotty pattern of the NiCo2W phase changed here to a smooth pattern of fine-grained NiCo2W 111 reflection on the right side of the angular range while the character of the W-B2 110 reflection (on the left) remained unchanged after rotary swaging.

The finding of the large-grain microstructure of the NiCo2W phase in the W_0 sample bar stressed the necessity to rotate the samples around the vertical axis in order to minimize the influence of the large grains on the resulting diffractogram and consequently on the microstrain determination.

**Figure 3:**
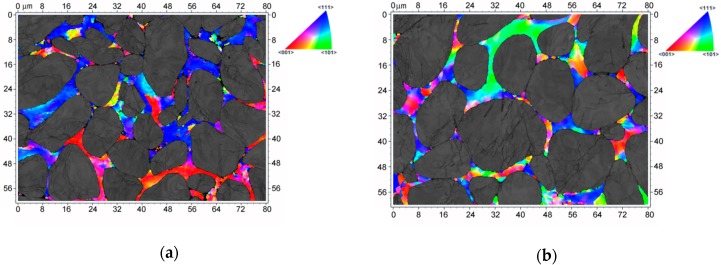
Electron backscatter diffraction (EBSD) scan of the surface perpendicular to the sample-bar axis depicting NiCo2W phase for sample: (**a**) W_A; (**b**) W_B. The depicted colors in the unit triangle indicate the orientation of the axis normal to the investigated sample surface in the crystal reference frame. The gray areas are W-B2 agglomerates.

The results of neutron diffraction analyses were further supported via electron backscattering observations. Figure 3a,b shows EBSD scans depicting the orientations of the NiCo2W grains in W_A and W_B samples, respectively. The depicted colours in the unit triangle indicate the orientation of the axis normal to the investigated sample surface in the crystal reference frame. With respect to the W_0 sample (not shown here), the size of NiCo2W grains was significantly refined and substructure developed. The average NiCo2W grain sizes were 1.35 µm for the W_A sample and 1.0 µm for the W_B sample. Regarding the W-B2 grains, the original W powder agglomerated during sintering and formed particles with the sizes of several dozens of micrometres, as can be seen in Figure 3 and as was also reported previously [6].

It comes from the concurrent texture investigation by neutron scattering, which will be published elsewhere, that there was no preferential orientation of the NiCo2W phase in the W_0 bar. On the other hand, the NiCo2W phase was textured with <111> crystallographic directions preferentially oriented along the sample bar axis after rotary swaging. The texture was significantly stronger for the cold swaged sample W_A than for the warm swaged sample (W_B). There is a certain relationship between the texture and mechanical properties. Nevertheless, the relationship is not described here purposely, as it will be a topic of a detailed study published in a future paper.

### 3.2. Data for Microstrain Determination

**Figure 4:**
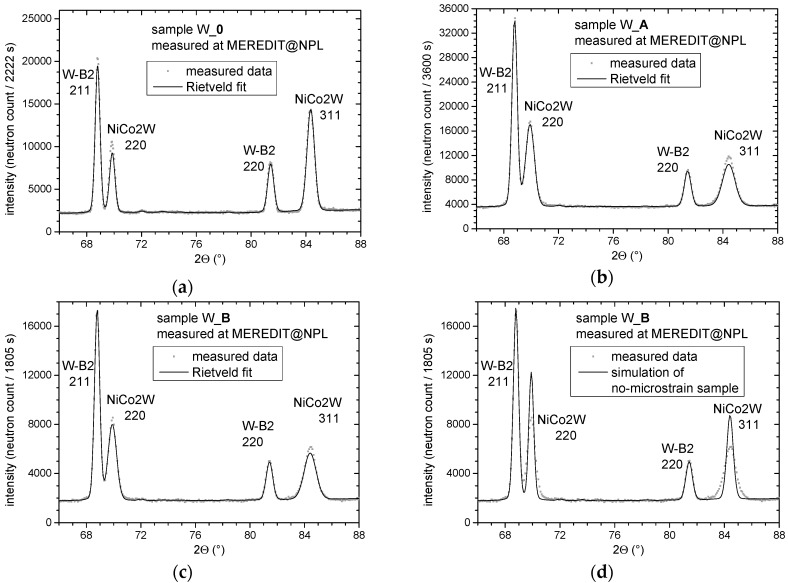
Detail of measured and calculated diffractograms showing the same indexed reflections from both W-B2 and NiCo2W phases in (**a**) W_0, (**b**) W_A and (**c**) W_B samples. (**d**) Additional W_B sample data together with theoretical simulation of a sample profile without any microstrain present in the NiCo2W phase (i.e., only the instrumental broadening effect is present).

Figure 4a–c display the zoomed selected angular range of the measured and calculated diffractograms for all three samples with indexed reflections for both W-B2 and NiCo2W phases. In order to display the extent of sample peak broadening, Figure 4d additionally shows W_B sample data together with a pattern calculated without any sample broadening effect.

By comparing the measured peaks (see Figure 4) of the W-B2 phase for the sample without deformation and samples after the RS process, no significant changes are recognized. On the other hand, NiCo2W reflections broadened significantly with respect to pure instrumental broadening (see Figure 4d). This indicates an increase in microstrain and dislocation density in NiCo2W phase while the W-B2 phase was not affected significantly.

The microstructural fit to the measured data is discussed in Section 4.

### 3.3. Electron Microscopy

**Figure 5:**
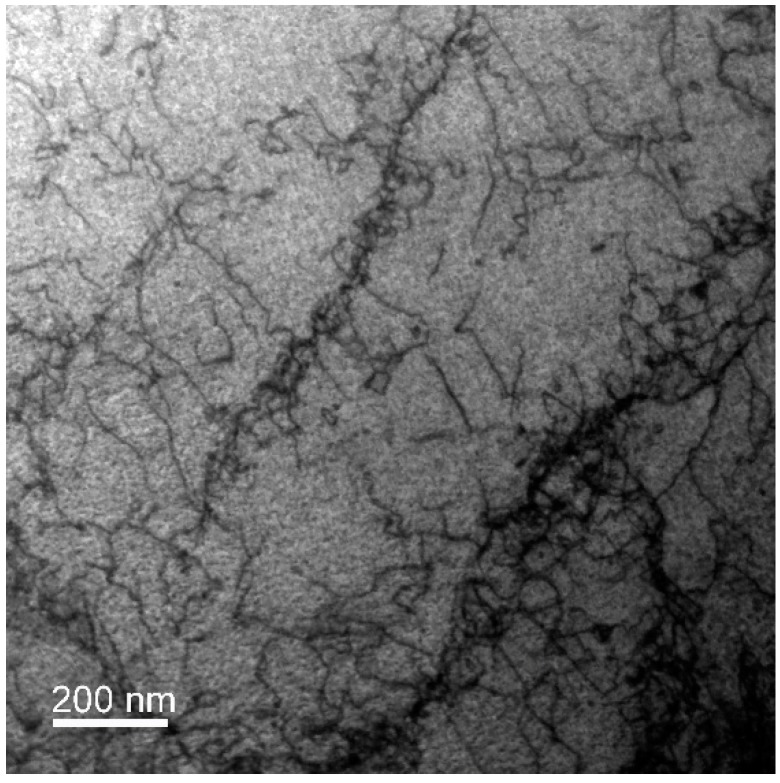
Transmission electron microscope image of W_A sample NiCo2W phase.

Figure 5 shows a TEM scan of the W_A sample taken in a location near the NiCo2W/W-B2 interface. The substantial presence of dislocations within the NiCo2W matrix was observed.

### 3.4. Material Properties

**Figure 6:**
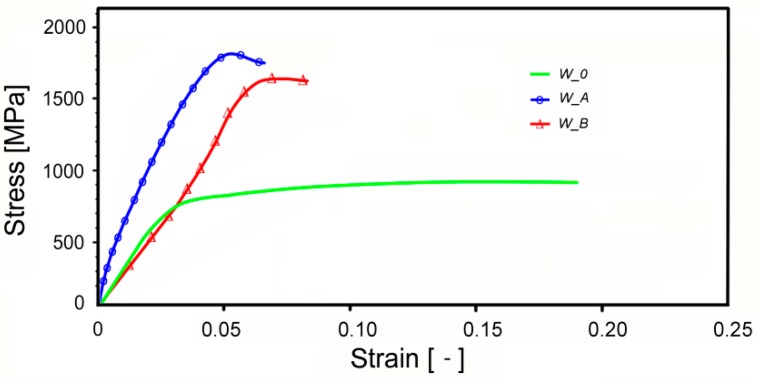
Experimental stress-strain curves for W_0, W_A, and W_B samples.

The stress-strain curves for W_0, W_A, and W_B samples are depicted in Figure 6. The sintered W_0 sample exhibited the lowest UTS of approximately 860 MPa. On the other hand, the sample featured a relatively high maximum elongation of more than 18%. As a result of the intensive imposed shear strain, the strength increased substantially; however, plasticity (maximum elongation) decreased after both swaging regimes. The total strengthening, i.e., UTS, was higher for the W_A sample, whereas the W_B sample featured higher plasticity.

The physical properties measured via ultrasound are depicted in Table 1 (the average value from 5 independent measurements taken per sample).

## 4. Data Analysis and Discussion

Detailed analysis of the measured neutron diffraction data was carried out with the intention to determine microstrain, dislocation type and dislocation density. With the support of the data obtained by other techniques, these microstructural parameters are related to the determined mechanical properties.

### 4.1. Microstrain Determination

First, a phenomenological approach was used to determine microstrain and the line integral breaths of all the measured reflections. FullProf software [30] enables the fitting of a phenomenological model of peak broadening caused by microstructural features, particularly microstrain and grain size, provided that the instrumental broadening is known across the whole measured 2θ range. The instrument profile dependency on scattering angle was obtained by measuring and fitting the standard SiO_2_ powder sample in the identical instrument setup. The profile function parameters were extracted and stored in the instrument resolution file and this file was used during structural and microstructural full-pattern refinement. Then, the FullProf refinement results in the sample contribution to the reflection broadening. This sample broadening of the diffraction peaks is reported in the further text.

The reflection profiles of the tungsten grains (W-B2 phase) in the W_0 sample exhibited no sample broadening. Therefore, no measurable microstrain is present for this phase in the sintered sample. The small broadening of diffraction peaks in the W-B2 phase after rotary swaging was observed. As the W-B2 phase grains were sufficiently large, no grain-size broadening was present. The sample broadening of the W-B2 peaks was then satisfactorily fit using isotropic microstrain. The output was the maximum (upper limit) strain *e* [31] which in fact represents the microstrain as it is connected with the root mean square strain (RMSS, $\langle \varepsilon_{0}^{2}\rangle {}^{1/2}$) through a constant scaling factor, $\langle \varepsilon_{0}^{2}\rangle {}^{1/2}=\sqrt{2/\pi }e$. The determined W-B2 phase upper limit strain for the W_A and W_B samples was *e*_W_A_ = 12.4 × 10^−4^ and *e*_W_B_ = 10.8 × 10^−4^, respectively. It can be seen that there is slightly higher microstrain in the W_A sample than in the W_B sample.

In the case of the NiCo2W phase, peak broadening was already present in the W_0 sample. After rotary swaging, the sample broadening effect still significantly increased. Although RS procedure refined the NiCo2W grains, they still remained sufficiently large in the measured samples (see Figure 3). The grain-size broadening was thus not expected. As the reflections from (200) family were visibly more broadened, the anisotropic strain broadening using Stephens formalism [32,33] was used to determine the microstrain contribution. The fit was successfully carried out assuming only microstrain broadening (i.e., no size broadening). Gaussian profile, as usually done for microstrain broadening, was used to satisfactorily describe the sample broadening effect.

**Figure 7:**
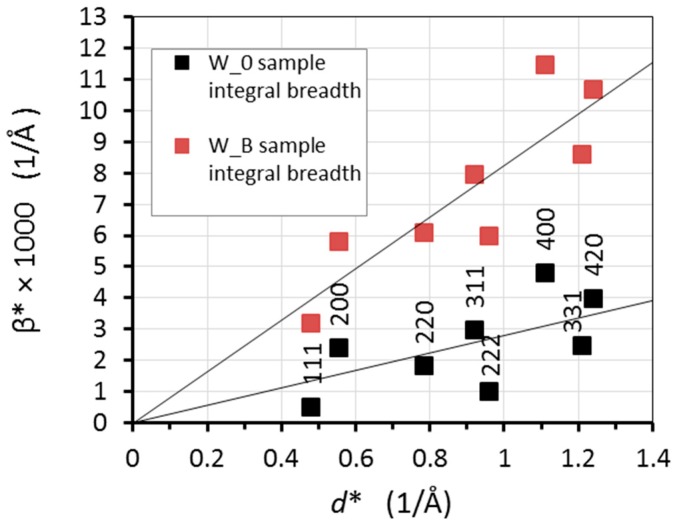
Williamson-Hall plot of integral breadths in reciprocal space for W_0 and W_B samples shown with linear fits through the points.

The outputs from the refinement using the anisotropic strain broadening are values of integral breadth for the individual reflections of the NiCo2W phase in reciprocal space, β* = (β cosθ)/λ, where β is the integral breath in 2θ scale, and also microstrain values for the measured diffraction peaks of the NiCo2W phase in all three samples. The sample integral breadth β* of each NiCo2W reflection is plotted as a function of reciprocal lattice spacing of the particular reflection d* in Figure 7 (classical Williamson-Hall plot [31]), for W_0 and W_B samples.

When considering only the strain broadening component as mentioned above, the upper limit strain *e* is connected with the integral breath by the formula β* = 2 ed*, i.e., its average value can be calculated from the slope of the linear dependence of β* on d* [31]. The linear fits through the points are shown in Figure 7. The determined average sample microstrain for the individual W_0, W_A, and W_B samples is then *e*_NiCo_0_ = 14.2 × 10^−4^, *e*_NiCo_A_ = 44.2 × 10^−4^ and *e*_NiCo_B_ = 41.2 × 10^−4^, respectively.

It can be seen that the microstrain in NiCo2W phase very significantly (approximately 3 times) increased after rotary swaging. Further, there is a slightly higher NiCo2W-phase microstrain in the W_A sample than in the W_B sample. Most probably, swaging at the temperature of 900 °C (W_B sample) enabled a partial dynamic recrystallization of NiCo2W phase.

The anisotropic character of the NiCo2W phase integral breadths, visible in the Williamson-Hall plot (Figure 7), indicates that microstrain is caused by dislocations. Further, the substantial presence of dislocations in the NiCo2W matrix was confirmed by TEM observations (Figure 5). Therefore, the data (i.e., acquired integral breaths for the NiCo2W phase individual reflections) were further analyzed in order to determine the active slip systems and to estimate the dislocation density after the thermomechanical processing.

### 4.2. Dislocation and Slip System Type

The fact that dislocation line broadening is usually anisotropic, i.e., depends on *hkl* reflection, is well known (see [34] and references therein). It is given by the anisotropic characters of the displacement fields of dislocations (line defects), which, moreover, are different for different types of dislocations and slip systems. Then, the anisotropy analysis can be in principle used to characterize the particular types of occurring dislocations and activated slip system [35]. Therefore, we tested this possibility also in the NiCo2W phase for the sintered and rotary swaged samples.

The anisotropy is characterized by the dislocation average contrast factors $C_{\alpha -hkl}$, which can be calculated with help of the ANIZC program [36] for various types of dislocations of given characters and slip system α and for all the measured *hkl* reflections. The calculations (i.e., determination of the average contrast factor for the possible types of dislocations) were performed for the measured NiCo2W reflections. To perform the calculations, elastic constants of the material were needed [37]. In order to use as precise values of the elastic constants for the NiCo2W solid solution as possible, the assumption of a linear combination of the individual elastic constants of the original constituents, i.e., Ni and Co, present in the ratio of 6:1, was made. The considered values found in the literature were the following: nickel [38]—C_11_ = 256.5 GPa, C_12_ = 151.5 GPa, C_44_ = 123.9 GPa; β-cobalt (fcc) [39]—C_11_ = 239.8 GPa, C_12_ = 163.4 GPa, C_44_ = 133.4 GPa. Their linear combinations used to estimate the elastic constants of the alloy were then: C_11_ = 246.9 GPa, C_12_ = 156.1 GPa, C_44_ = 125.5 GPa.

**Figure 8:**
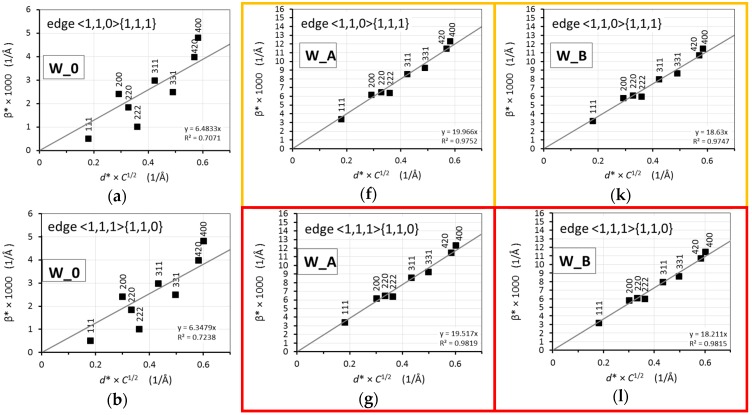

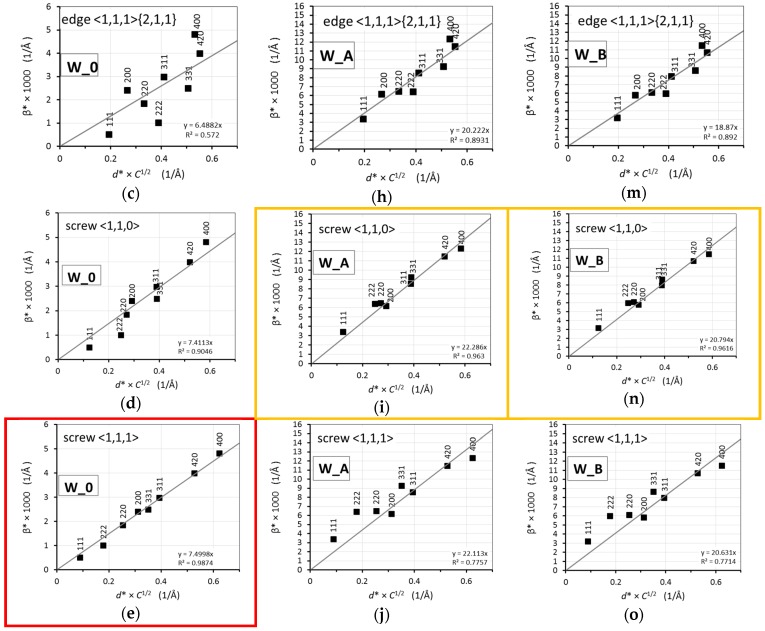
Modified Williamson-Hall plots considering various dislocation types and slip systems for samples: (**a**–**e**) W_0; (**f**–**j**) W_A; and (**k**–**o**) W_B. The red color framed plots represent the best-fitting model, the orange ones represent sufficiently good fits. These dislocation types and slip systems are considered as the most probable for the NiCo2W phase of the pseudoalloy and are used for a further evaluation (see text).

After calculation of the average contrast factors $\bar{C_{hkl}^{\alpha }}$, a modified Williamson-Hall plot [37], i.e., the dependence of β* on d*C^1/2^, can be drawn. The results for the W_0, W_A, and W_B samples are depicted in Figure 8a–o. As can be seen, the best result (concerning fitting the linear dependence) for the W_0 sample was acquired for screw dislocations and <111> slip system (Figure 8e; the corresponding modified Williamson-Hall plot is marked by a red frame). All the other tested dislocation types and slip systems were far worse.

On the other hand, the slip system of <111> {110} edge dislocations fits the best to the measured integral breadths of W_A and W_B samples (Figure 8g,l, respectively, marked by a red frame). Nevertheless, the edge dislocations with <110> {111} slip system (Figure 8f,k), as well as the screw dislocations with <110> slip system (Figure 8i,n), exhibited very good linear fits for the rotary swaged samples, too. The two remaining tested dislocation types and slip systems (screw dislocations and <111> slip system, and edge dislocations with <111> {211} slip system) did not exhibit a sufficiently good fit for any of the investigated samples.

Obviously, certain microstrain was present within the structure of the NiCo2W phase already after sintering and subsequent cooling. The dislocations produced during the sintering/quenching process were predominantly of a screw type with <111> slip system. After rotary swaging (W_A and W_B samples), the microstrain increased significantly (~3 times) and the deformation mechanism changed either to edge dislocations with <111> {110} or <110> {111} slip system (the latter occurs typically in fcc structures [40]), or to screw dislocations with <110> slip system.

**Figure 9:**
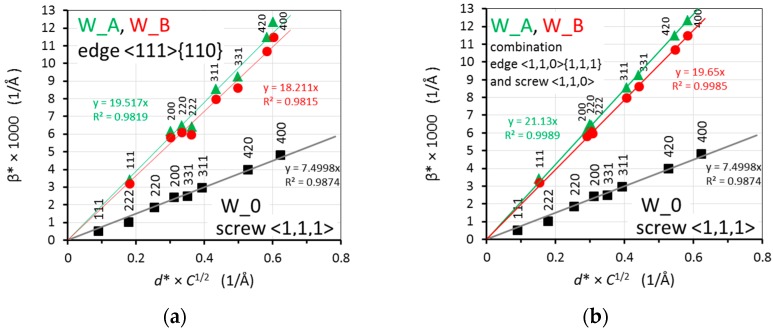
Summarized modified Williamson-Hall plots of integral breadths for W_0, W_A, and W_B samples shown with linear fits through the points: the edge dislocation with <1,1,1> {1,1,0} slip system for W_A and W_B samples (**a**); 50%:50% combination of edge dislocations with <110> {111} slip system and screw dislocations with <110> slip system (**b**).

Figure 9a then summarizes the modified Williamson-Hall plots of integral breadths for the best fitting slip systems for W_0, W_A, and W_B samples (edge dislocations with <111> {110} slip system for W_A and W_B samples). It should be stressed that the quality of fitting for the edge dislocations with <110> {111} slip system and screw dislocations with <110> slip system was almost equal (see Figure 8). A combination of edge dislocation with screw dislocation system was tested as well for W_A and W_B samples. The result for the combination of edge dislocations with <110> {111} slip system and screw dislocations with <110> slip system (the ratio of influence on integral breadth was assumed to be 50%:50%) is shown in Figure 9b. A very similarly good result (not shown here) was obtained for the 50%:50% combination of the edge dislocation with <1,1,1> {1,1,0} slip system and the screw dislocation with <1,1,0> slip system.

### 4.3. Dislocation Density

Information on dislocation types, slip systems and also on dislocation densities may be very useful for modelling the material behavior using crystal plasticity [41]. Based on the results of line broadening of the NiCo2W phase, dislocation densities *ρ* can in principle be estimated using various numerical procedures [34,35,42,43,44,45,46].

Powder diffraction techniques, in general, have limited accuracy in determining dislocation densities. Precise descriptions of the instrument profile function and the size of the instrumental broadening are essential for this purpose. However, even for precisely determined instrument parameters, another important aspect is the source intensity and the peak-to-background ratio, which are usually limiting in the case of neutron diffraction. Thus, only a rather limited accuracy on the absolute scale of the estimated dislocation densities can be achieved using the above-mentioned analysis of the neutron diffraction line broadening for the NiCo2W phase. Nevertheless, such information has an important indicative value when comparing relatively the obtained dislocation densities for the individual samples.

For the estimation of the density, we used one of the approaches presented in [35]. The integral breath of each reflection can be approximated by,

(1) $$\beta_{hkl}^{*}=\frac{2\sqrt{2}\;{\left(\ln P\right)}^{\frac{3}{2}}}{4\ln P-\ln\left(\ln P\right)}\;d_{hkl}^{*}C_{hkl}^{\frac{1}{2}}\;b\;\rho^{\frac{1}{2}}$$

where *b* is Burgers vector, and factor *P* is related to the correlation in the dislocation arrangement.

Assuming that the dislocation correlation factor *P* [35] can be reasonably estimated, dislocation density *ρ* can be determined based on Equation (1). Due to the moderate resolution of the diffraction data, *P* could not be determined directly from the diffraction profile analysis. For further calculations, the value 10 for the *P* factor was used which is a reasonable value for Gaussian profiles [35] employed in our analysis of the strain broadening. The estimated error in absolute value for *ρ* was −20% (in this case, *P* would be equal to 15) and +50% (in this case, *P* would be equal to 5).

Considering the above-mentioned assumptions, the dislocation densities for the most favorable slip system in NiCo2W for all the investigated samples were calculated and they are listed below:

W_0: 1.7 × 10^11^ cm/cm^3^ (screw dislocations with <111> slip system);
W_A: 8.7 × 10^11^ cm/cm^3^ (either edge dislocations with <110> {111} slip system or <110> screw dislocations);
W_B: 7.6 × 10^11^ cm/cm^3^ (either edge dislocations with <110> {111} slip system or <110> screw dislocations).

As explained above, absolute values of dislocation densities are burdened by a large error. Nevertheless, a relative comparison of the obtained values of dislocation densities between the individual samples brings significantly more precise information as the relative error of the dislocation density values is expected to be similar for all the samples. Then, it can be seen that the dislocation densities increased approximately 5 times after rotary swaging, and that the dislocation density is 15% higher for the sample swaged at room temperature than for the sample deformed at 900 °C.

### 4.4. Mechanical Properties

The measured stress-strain curves (Figure 6) are in accord with the results of the microstructural study. Relatively low UTS and high maximum elongation for the sintered W_0 sample correspond to the coarse grain size of the NiCo2W phase, which, together with the low dislocation density, did not ensure sufficient strengthening.

The plasticity (maximum elongation) decreased after both swaging regimes. Nevertheless, the intensive imposed shear strain imparted significant accumulation of dislocations in the NiCo2W phase and the strength of the material increased substantially. The accumulated dislocations after RS provide hardening to the NiCo2W phase and thus enable effective transfer of the imposed strain to the W-B2 phase. Although the imposed deformation is predominantly accumulated in the NiCo2W phase, it does not mean that the W-grains are not strained at all. Tungsten grains deformation was already observed in the past [19,20,47]. Also in this study, a small level of microstrain within the W-B2 phase in the rotary swaged samples was detected (see Section 4.1).

The W_A sample, featuring the highest dislocation density and notable presence of microstrain in the NiCo2W phase, exhibited higher total strengthening (i.e., UTS), whereas the W_B sample featured higher plasticity. Mutual comparison of the stress-strain curves for the W_A and W_B samples also revealed a more gradual strengthening (i.e., smoother curve shape) of the W_B sample. Swaging at the temperature of 900 °C enabled the NiCo2W phase to dynamically recrystallize, which was documented by the very small average grain size of 1.0 µm (Section 3.1) and decreased microstrain values (Section 3.2), and provided it with the ability to consume a greater amount of the imposed energy. This finding is in accordance with the results documented by Katavič et al. [48] who reported the hardening rate of the matrix during intensive shear deformation of tungsten pseudoalloys to be more than two times higher than the hardening rate of W particles up to approximately 15% deformation.

## 5. Conclusions

This study focused on characterization of the effects of rotary swaging at various temperatures on a WNiCo tungsten heavy alloy via determination of microstrain and characterization of dislocation types and activated slip systems.

The results showed that the original sintered sample consisted of fine-grained spherical W-B2 type agglomerates surrounded by a coarse-grained NiCo2W matrix. The W-B2 agglomerates did not feature any significant microstrain. However, the NiCo2W matrix exhibited microstrain (magnitude 14.2 × 10^−4^) resulting mainly from the presence of screw dislocations with <111> slip system.

Both cold and warm rotary swaging then imparted grains fragmentation for the NiCo2W matrix and resulted in formation of fine-grained structures within the NiCo2W phase. From results presented elsewhere [49,50], it is also clear that the W-B2 phase is refined by rotary swaging. Further, neutron diffraction revealed that the microstrain increased three times in the NiCo2W phase (44.2 × 10^−4^ and 41.2 × 10^−4^ for W_A and W_B samples, respectively). On the other hand, a rather low level of microstrain was detected in the tungsten W-B2 phase of the composite after the rotary swaging.

The measured mechanical parameters correspond to the results of the microstructural characterization. The swaged samples exhibited substantial strengthening which was primarily caused by the increase in dislocation density (~5× for the 900 °C sample, and even approximately 10% more for the cold swaged one) in the NiCo2W phase. The 20 °C swaged bar featured the ultimate tensile strength of almost 1900 MPa. It can be concluded from the neutron diffraction that the dominant deformation mechanisms for both the 20 °C and 900 °C rotary swaged samples were edge dislocations with <111> {110} or <110> {111} slip system, or screw dislocations with <1,1,0> slip system in NiCo2W phase. A combination of the above-mentioned systems is most probable for the NiCo2W phase as these combinations lead to the best modified Williamson-Hall plot.

## Figures and Tables

**Table 1:** Physical properties resulting from ultrasound measurements.

Sample	Young’s Modulus (GPa)	Shear Modulus (GPa)	Poisson’s Ratio (-)
W_0	340	130	0.280
W_A	350	137	0.278
W_B	359	141	0.270
